# Intra-articular knee arborescent lipoma: a case treated with arthroscopic synoviectomy

**DOI:** 10.11604/pamj.2017.28.25.12800

**Published:** 2017-09-13

**Authors:** Aymen Saidi, Lassaad Hassini, Aymen Fekih, Monia Ben Othmen, Mohamed Allagui, Abderrazek Abid, Issam Aloui

**Affiliations:** 1 Department of Orthopaedic Surgery, University Hospital, Monastir, Tunisia

**Keywords:** Arborescent lipoma, knee, synoviectomy, arthroscopic

## Abstract

Arborescent lipoma is an unusual intra-articular lesion that typically develops in the knee and has to be evoked before chronic effusion. It corresponds to hyperplasia of mature fatty tissue and hypertrophy of synovial villi, developing within a joint. The reference treatment is synovectomy by arthrotomy. The rare forms localized to the anterior compartment of the knee can benefit from an arthroscopic synovectomy. The authors report a case of arborescent knee lipoma in a 47-year-old patient who received arthroscopic synoviectomy. To our knowledge, only a few cases of arborescent lipoma treated by arthroscopic synoviectomy have been reported in the literature.

## Introduction

The arborescent lipoma of the knee is a rare synovium pseudotumor, of unknown etiology. It is composed of hypertrophied synovial villi and containing a very large amount of fat, so that the mass has a lipomatous aspect [1]. Its association with osteoarthritis, rheumatoid arthritis or an old trauma has been reported [2–4]. This pseudotumor is accompanied by symptoms that have lasted for months and even years and a painless and recurring synovial effusion. The adapted sequences of Magnetic Resonance Imaging can confirm the fatty nature of proliferation. We report a case of arborescent knee lipoma in a 47 year old patient treated with arthroscopic synovectomy.

## Patient and observation

**Figure 1:**
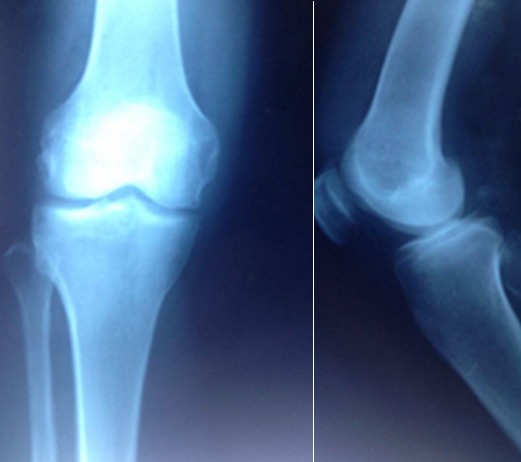
Frontal and profile right knee radiography showing internal stage 2 femoro-tibial knee osteoarthritis

**Figure 2:**
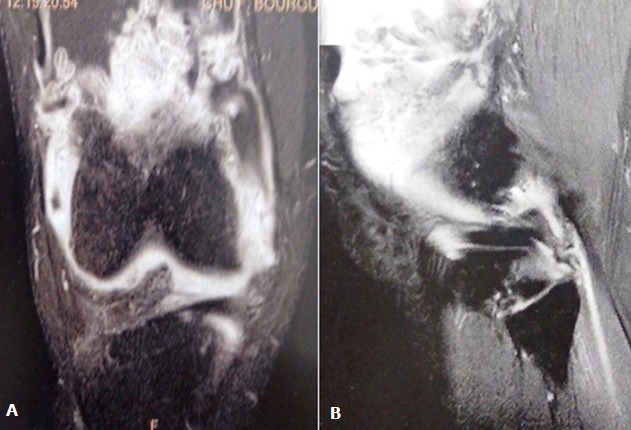
Magnetic resonance imaging: the frontal (A) and sagittal (B) sections of the right knee showing hyper-signal synovial proliferation which predominates at the quadriceptal recess

**Figure 3:**
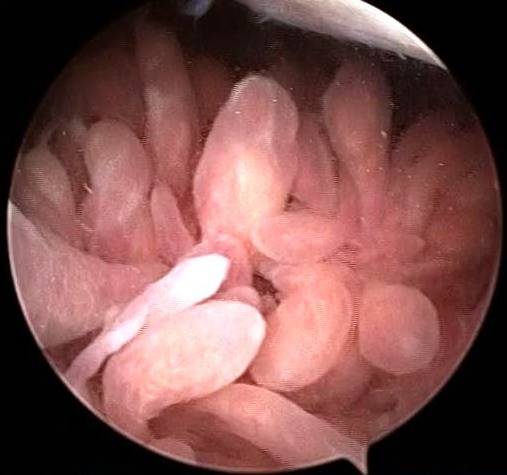
Arthroscopic view of the knee showing synovial hyperplasia with yellowish-white digitization

**Figure 4:**
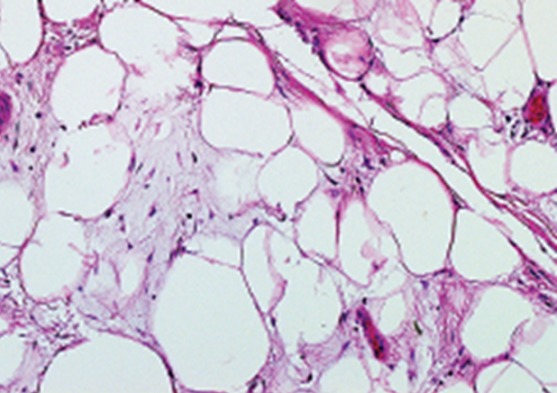
Histology of the synovial biopsy showing an adipocyte proliferation in the synovium

It is a 47-year-old patient with no pathological history, with disabling mechanical pain in the right knee, progressively progressive for 18 months, resistant to analgesic treatment and knee swelling that gradually increased in volume. This case was evolving in a context of apyrexia and conservation of the general state. The clinical examination found an articular effusion and a swelling of the sub-quadricipital recess. The standard X-ray showed internal stage 2 femoro-tibial knee osteoarthritis (Figure 1) and the usual biological examinations were without abnormalities. The knee puncture fluid was pale yellow, sterile and crystal-free. Magnetic Resonance Imaging (MRI) evoked the diagnosis of a knee lipoma arborescent by showing synovial proliferation at the level of the sub-quadricipital recess associated with an effusion of the knee (Figure 2). Arthroscopy of the knee was performed and a yellowish-white synovial hyperplasia was detected at the sub-quadricipital recess and the condylar ramps (Figure 3). Arthroscopic synoviectomy was performed at the same operative stage in the anterior knee compartment. Microscopic examination showed that the synovium was infiltrated by mature adipocytes with hyperplasia of adipose tissue (Figure 4). At 18 months follow-up, there was no clinical recurrence and the functional discomfort was discreet.

## Discussion

Arborescent lipoma or diffuse synovial lipoma is a rare fatty pseudotumor (0.3 to 0.7% of lipomas), corresponding to hyperplasia of mature fatty tissue and hypertrophy of synovial villi, developing within a joint [4]. It is most often located at the knee joint, in the sub-quadricipital recess. Its association with osteoarthritis, rheumatoid arthritis or an old trauma and the histological evidence of chronic synovial inflammation, suggests a non-specific reaction origin rather than a neoplastic origin [2-4]. Hallel proposed to evoke “lipomatous villous proliferation of the synovial membrane” rather than of arborescent lipoma [5]. It is found particularly in men of the 4^th^ and 5^th^ decades and can develop at any age. The diagnosis of the Arborescent Lipoma is difficult because the symptoms are not very specific and may be present in many other pathologies. This lesion is clinically indicated by a somewhat mechanical discomfort or pain, evolving for many years with periods of exacerbation, probably related to the underlying arthropathy or sometimes by an anterior articular or para-articular knee swelling of progressive, little painful onset and often associated with painless and recurrent joint effusion [5]. The arborescent lipoma may also be discovered during spontaneous hemarthrosis, especially in young subjects [4]. If X-rays may suggest intra-articular hypodensity, it is mainly the ultrasound which shows the effusion and the villous character of the mass. CT and even better MRI, can be used to confirm the diagnosis [6,7]. The arborescent lipoma results in fatty hypertrophy of the synovial fringes and, more rarely, a sub-synovial fatty mass. The lesion signal is identical to that of the adjacent fat on all sequences. A hyper signal T2 and an enhancement of certain non-fatty lipoma components are possible after injection of gadolinium and should not cause worry (associated synovitis). There are no hemosiderin deposits. An intra-articular effusion is frequently associated with possible artifacts of chemical shift at the interface between the lipoma and the synovial fluid. Differential diagnosis is mainly due to villonodular synovitis, which has different MRI characteristics due to signal abnormalities associated with hemosiderin deposits. Arthroscopy shows a white-yellow synovial proliferation forming variable size fingerings, giving a tree-like appearance [8]. The anatomopathological examination confirms the diagnosis by showing a hyperplasia of the adipose tissue extending to the contact of the synoviocyte coating. The reference treatment is synovectomy after arthrotomy. The arthroscopic treatment of the forms localized to the anterior compartment of the knee is proposed by Blais et al and Sola et al [8, 9]. To our knowledge, only a few cases of arborescent lipoma treated by arthroscopic synoviectomy have been reported in the literature. The prognosis is favorable after treatment and there is usually no recurrence after synovectomy [4]. Recurrence occurs if the therapeutic gesture is incomplete. Nisolle et al. reported a case of arborescent knee lipoma treated by chemical synovectomy with osmic acid which has been reported with good results at one year follow-up [10].

## Conclusion

The Arborescent Lipoma of the knee is a rare condition that must be evoked before a chronic articular effusion. Magnetic Resonance Imaging (MRI) makes it possible to evoke the diagnosis by showing the greasy nature of the synovial proliferation and specifies its extension. The arthroscopic synoviectomy in the localized forms at the anterior compartment of the knee is an alternative to arthrotomy.

## Competing interests

The authors declare no competing interests.
